# The interplay between climate change and ageing: A systematic review of health indicators

**DOI:** 10.1371/journal.pone.0297116

**Published:** 2024-04-24

**Authors:** Teodora Figueiredo, Luís Midão, Pedro Rocha, Sara Cruz, Gisela Lameira, Paulo Conceição, Rui J. G. Ramos, Luísa Batista, Helena Corvacho, Marta Almada, Ana Martins, Cecília Rocha, Anabela Ribeiro, Fernando Alves, Elísio Costa

**Affiliations:** 1 Porto4Ageing—Competence Center on Active and Healthy Ageing of the University of Porto, Faculty of Pharmacy of the University of Porto, Associate Laboratory i4HB—Institute for Health and Bioeconomy and UCIBIO—Applied Biomolecular Sciences Unit, Faculty of Pharmacy of the University of Porto, Porto, Portugal; 2 CINTESIS@RISE, “Department of Behavioral Sciences”, ICBAS, University of Porto, Porto, Portugal; 3 CITTA–Research Centre for Territory, Transports and Environment, Department of Civil Engineering, Faculty of Engineering of the University of Porto, Porto, Portugal; 4 Faculty of Architecture, University of Porto, Porto, Portugal; 5 CONSTRUCT (LFC), Faculty of Engineering University of Porto, Porto, Portugal; 6 CITTA–Research Centre for Territory, Transports and Environment, Department of Civil Engineering, Faculty of Sciences and Technology of the University of Coimbra, Coimbra, Portugal; Menzies School of Health Research: Charles Darwin University, AUSTRALIA

## Abstract

Climate change and rapid population ageing pose challenges for communities and public policies. This systematic review aims to gather data from studies that present health indicators establishing the connection between climate change and the physical and mental health of the older population (≥ 65 years), who experience a heightened vulnerability to the impacts of climate change when compared to other age cohorts. This review was conducted according to the PICO strategy and following Cochrane and PRISMA guidelines. Three databases (PubMed, Scopus and Greenfile) were searched for articles from 2015 to 2022. After applying inclusion and exclusion criteria,nineteen studies were included. The findings indicated that various climate change phenomena are associated with an elevated risk of mortality and morbidity outcomes in older adults. These included cardiovascular, respiratory, renal, and mental diseases, along with physical injuries. Notably, the impact of climate change was influenced by gender, socioeconomic status, education level, and age—vulnerability factors. Climate change directly affected the health of older adults through ambient temperature variability, extreme and abnormal temperatures, strong winds, sea temperature variability, extreme El Niño-southern Oscillation (ENSO) conditions and droughts, and indirectly by air pollution resulting from wildfires. This review presents further evidence confirming that climate change significantly impacts the health and well-being of older adults. It highlights the urgency for implementing effective strategies to facilitate adaptation and mitigation, enhancing the overall quality of life for all individuals.

## Introduction

The average lifespan has been greatly extended due to advancements in nutrition, technology, and medicine. Along with this, the declining birth rates have contributed to a rise in the population of individuals aged 65 years or older [1]. In 2019, there were approximately 703 million people in this age group worldwide, which is projected to reach 1.5 billion by 2050, an overall increase from 9 to 16% [2]. As individuals age, physical and mental declines occur, making older adults more vulnerable to external factors such as extreme temperatures and air pollution. According to a report by the United Nations (UN), both ageing and climate change are current urgent global concerns [3]. Several organizations have been collaborating to find consensus and implement strategies for creating more sustainable and resilient societies and cope with the challenges of climate change. The Lancet Countdown, an international collaboration of 51 academic institutions and UN agencies, has been at the forefront of monitoring the health impacts of climate change. Their comprehensive research shows that the consequences of climate change extend beyond environmental issues and directly affect the health and well-being of populations, especially older adults. It emphasizes the need for a health-centered response to address the complex and interconnected challenges of our time, ensuring that older adults and other vulnerable populations are adequately protected from the adverse effects of climate change while fostering more sustainable and resilient communities [4].

In fact, climate change has far-reaching consequences for populations, affecting them in different ways depending on where they live. Vulnerable communities in low-income regions often bear the greatest burden of climate change, characterized by limited resources, insufficient infrastructure, and reduced adaptive capacity [5].

Older adults are especially vulnerable when it comes to mortality and morbidity. Studies show that heat-related mortality among older adults (aged 65 and over) has increased by 53.7% in recent decades [6]. Air pollution, as a driver/consequence of climate change, is responsible for 6.7 million deaths worldwide each year, making it the leading environmental cause of human morbidity and mortality. A quarter of these deaths occur in people over the age of 65, due to cardiovascular diseases [7, 8]. Climate change also affects cognitive function, and although the long-term effects are not yet well understood, there is a correlation between temperature variation and neurological diseases such as Alzheimer’s [9, 10]. It was already reported that older individuals with dementia or diminished cognitive function, coping abilities, and stress thresholds may struggle with environmental stressors, further exacerbating mental health conditions and increasing the risk of premature death [11]. It is important to emphasize that on a global scale, in 2016, neurological disorders stood as the foremost contributor to disability, accounting for 276 million disability-adjusted life-years, and ranked as the second leading factor responsible for mortality, causing 9 million deaths worldwide [12]. These climate changes have a range of adverse health impacts. Extreme heat can cause heat-related illnesses, worsen pre-existing conditions, and increase the risk of injury or death [13, 14]. Poor air quality can lead to respiratory and cardiovascular diseases, as well as other health problems [15]. Flooding can cause physical injury, water-borne diseases, and mental health problems, while vector-borne and water-related infections can lead to a range of illnesses and even death [16–20]. Food-related infections can also result in a higher burden on healthcare systems, with the associated hospitalizations, and even deaths [21, 22]. Furthermore, droughts can cause water shortages, which subsequently result in crop failures, economic downturns, and, consequently, the displacement of populations and even conflict [23]. Besides, climate change can also impact mental health, causing trauma, stress, anxiety, and social inequality, especially among vulnerable populations like those affected by extreme weather events and displacement [24, 25]. Populations that are most vulnerable to these health impacts include older adults, young children, outdoor workers, and individuals with chronic medical conditions, weakened immune systems, limited access to healthcare, and inadequate access to clean water and nutritious food. Climate change exacerbates these vulnerabilities, highlighting the urgent need for adaptation and mitigation strategies to address these health impacts [26]. The urgency of addressing healthcare solutions in light of climate change has significantly heightened. Age, together with gender, low socioeconomic status, social isolation and low level of education, are some of the factors that increase individuals’ and groups’ vulnerability, with the older population being a prime example [27]. Additionally, there are psychological factors that can modulate individuals’ vulnerability to climate change. For instance, the psychological distance factor. This implies that people tend to perceive climate change as a more immediate concern when they see it as closer to them in both time and place. This heightened sense of closeness often results in a greater willingness to embrace pro-environmental and adaptive behaviours. Conversely, when individuals perceive climate change as more distant in time or place, it tends to create a more abstract representation of the issue [28, 29].

Literature has descriptively presented the challenges that relate to climate change and the implications for the health of older adults—in its different manifestations. Limitations such as small sample size studies or limited data availability usually restrict the statistical power and generalizability of the findings. Additionally, information is still scarce, and further research is needed, with regard to measurable indicators related to the impact of certain phenomena (heat waves, intense rains, air pollution, among others) on the physical and mental health of older adults [11, 30–33].

The studies that provide concrete indicators of the impact of climate change on the health of old adults give further information for the definition of public policies aimed at adapting public spaces, developing information and training processes, as well as promoting access to mechanisms that mitigate exposure to risk factors such as variations in temperature. Thus, it is also important to have a better awareness of the relationship between ecological, socioeconomic and health determinants to consider about the built environment in order to adapt to climate change, ensuring a better quality of life for all citizens particularly, the most vulnerable such as the older adults [30, 34].

To our knowledge, and to date, there are no systematic reviews exploring global climate change scenarios and their corresponding health outcomes in older people. Therefore, the objective of this systematic review is to collect quantifiable health indicators that establish a connection between climate change, the older population, and their physical and mental health. This review endeavors to furnish a substantial level of evidence and benefit research groups, policymakers, funding agencies, and other stakeholders involved in this field. By providing these professionals with prompt access to the most recent information in patient healthcare management, this review aims to support their practice and assist in the development of future research programs and trials.

## Materials and methods

The methodology employed for this systematic review adhered to the guidelines outlined in the Cochrane Handbook for Systematic Reviews of Interventions [35].

In order to ensure that this systematic review effectively addresses a relevant question and provides meaningful benefits to the scientific and healthcare community, the input of experts with diverse and noteworthy backgrounds was sought throughout the entire process—from identifying the review question to selecting the final articles. The protocol for this systematic review is registered on PROSPERO under the registration number CRD42022366182.

### Research question

Using the PICO strategy [36] and following Cochrane guidelines [35], the question of this review was: How do climate changes affect the physical health and well-being of the older population?

### Review objective

The objective of this review was to identify health indicators that elucidate the unidirectional association between climate change and the physical and mental health of the older population.

### Data collection

In March 2023, two reviewers, T.F. and L.M, independently searched and extracted data from PubMed, Scopus and Greenfile. The final search query was then constructed as: (‘old*’ OR ‘geriatr*’ OR ‘aged’ OR ‘ageing’ OR ‘aging’ OR ‘later life’ OR ‘elder*’) AND (‘individual health’ OR ‘health’ OR ‘normalit*’ OR ‘normalc*’ OR ‘illness’ OR ‘disease’ OR ‘condition’) AND (‘climate change’ OR ‘global warming’ OR ‘sea level rise’ OR ‘climate adaptation’). Language restrictions were applied to include only studies published between 2015 and 2022, in English. Besides, the search was restricted only to studies about humans.

All 3154 articles were reviewed by the same reviewers, who independently assessed the titles, in the first phase, then the abstracts, and finally the full text. The inclusion and exclusion criteria used throughout the selection process are described below.

### Inclusion and exclusion criteria

When assessing the titles, articles that met one or more of the exclusion criteria listed below were not considered:

1. topics unrelated to health, climate change or/and older population;
2. subjects of the study were not humans;
3. not published in English.

During the abstract and full-text analysis, the selection was based on the following inclusion and exclusion criteria:

#### Inclusion criteria

1. Study design: Studies that report on original research in humans (e.g., randomized controlled trials, observational studies, case-control studies, cross-sectional studies).
2. Population: Studies focusing on adults 65 years and older.
3. Exposure: Studies that investigate the impact of climate change on health outcomes in older adults, such as heat-related illnesses, cardiovascular and respiratory diseases, mental health, and other related outcomes.
4. Outcome: Studies that report on climate change’s effects on older adults’ health and their health-related quality of life, specifically in terms of morbidity and mortality.
5. Publication: Studies published in peer-reviewed journals.

#### Exclusion criteria

1. Studies that do not focus exclusively on older adults (i.e., studies that include mixed populations without a focus on older adults).
2. Studies that do not investigate the impact of climate change on health outcomes.
3. Studies that focus on animal or laboratory-based research.
4. Studies that do not report original research (e.g., review articles, editorials, comments, letters).
5. Studies that focused on future climate change scenarios projections of climate change or health outcomes.

### Interrater reliability

Two reviewers were responsible for the data selection process, with a third reviewer (E.C.) resolving any discrepancies. The SPSS software v.28.0 (IBM Corp. Armonk, NY, USA) was employed to determine the level of agreement (interrater reliability) among the reviewers. The reliability of data collection is crucial, particularly in healthcare and clinical research studies, to ensure consistency when selecting articles for a systematic review or samples for a study. Cohen’s kappa statistics were utilized to measure the interrater reliability of the selected articles for this study [37]. The article selection process for this systematic review was conducted in three phases: title selection, abstract selection, and full article selection, with Cohen’s kappa value calculated independently for all phases.

## Results

**Fig 1:**
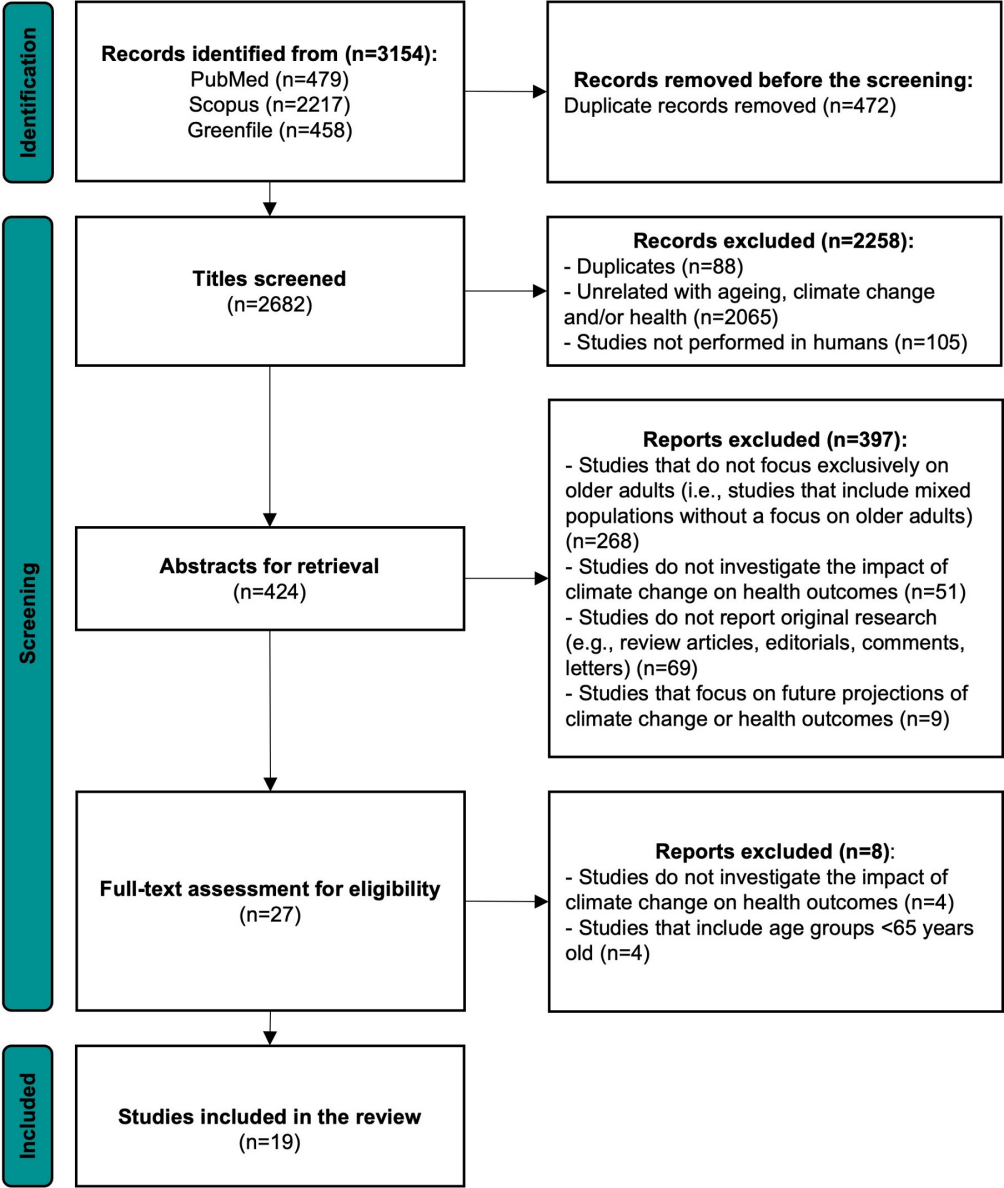
Articles’ screening process based on the PRISMA flow diagram.

The systematic search retrieved 3154 papers (Fig 1). In the end, after removing the duplicates and applying the above-mentioned inclusion and exclusion criteria, 19 studies were considered suitable for this systematic review and were analyzed to identify health indicators of the impact of climate change on older adults [10, 38–55]. After reviewing 2682 titles, 424 abstracts were identified for further analysis. Among these, 27 articles underwent full-text analysis, resulting in 19 articles being chosen for further examination. Cohen’s kappa values were independently calculated for each phase. A moderate kappa value of 0.553 was observed for inter-rater reliability during the title selection phase, while an almost perfect agreement was achieved during the abstract and full-text selection phases, with kappa values of 0.910 and 0.914, respectively.

Most articles were ecological studies (n = 8) [40, 42, 43, 45, 48, 53–55] and the rest were retrospective cohort studies (n = 5) [38, 39, 47, 51, 52], case-crossover studies (n = 3) [41, 46, 50], prospective cohort studies (n = 2) [10, 49] and a case-control study (n = 1) [44].

In the included articles, the locations studied were dispersed across North America [10, 38, 41, 43, 44, 46, 48, 51, 54], Asia [39, 42, 47, 49, 52, 53], Europe [40, 45, 50, 54], and Oceania [55], and none in Africa or South America. The majority of the included articles covered developed countries. The duration of the data collection ranged from 2 to 44 years. The studies investigated the effect of different climate change phenomena on the health of older adults, the most recurring being various forms of ambient temperature and its variability. Extreme temperatures were also a common exposure. Other climate change phenomena investigated included drought periods, air pollution from wildfires, strong winds, sea surface temperature variability and extreme El Niño-southern Oscillation (ENSO) conditions. The human health outcomes were related to mortality, cardiovascular, respiratory, renal, and mental health risks. One study also investigated hip fracture incidence. Table 1 summarizes the characteristics and main findings of the studies in this systematic review.

**Table 1:** Main findings and characteristics of the select articles.

Authors, Year	Study Design	Country (Data Collection Years)	Climate Change Phenomenon	Health Outcome	Indicators	
					Morbidity	Mortality
Berman *et al*., 2017 [38]	Retrospective cohort study	USA (2000–2013)	Full drought periods (≥150 days)	Respiratory-related hospitalization and all-cause mortality	During full drought periods, hospitalizations decreased by -1.99% (95% PI: -3.56,-0.38) vs non-drought periods.	Mortality risk increased by 1.55% (95% PI: 0.17–2.95) during high severity worsening drought periods.
Chen & Zhang, 2022 [39]	Retrospective cohort study	China (2002–2018)	Average annual T	Cardiovascular diseases (hypertension, heart disease and stroke)	Every 1°C increase in T reduced the rates of hypertension by 3% (OR: 0.97; 95% CI: 0.96–0.97), heart disease by 6% (0.92–0.95) and stroke by 5% (0.94–0.97).	NA
Donaldson *et al*., 2019 [40]	Ecological study	UK (1995–2016)	Cold (T < 18°C)	All-cause mortality	NA	Cold-related mortality of females (aged 85+) increased over 1995–2016 vs 1977–1994.
Gronlund *et al*., 2016 [41]	Case-crossover study	USA (1992–2006)	EH	Hospitalizations for renal, heat and respiratory causes	Two-day EH effects: hospitalizations increased by 8% (95% CI: 6–11). Six-day EH effects: hospitalizations increased by 11% (95% CI: 8–14).	NA
Lim *et al*., 2016 [42]	Ecological study	Japan (1966–2010)	Abnormal high T (measured by TDI)	All-cause mortality	NA	Average 0.5% (95% CI: 0.1–1.0) increase in mortality per 1-unit increase in TDI. On summer days with moderate temperature, the increase in mortality was 1.9% (95% CI: 1.1–2.6) per 1-unit increase in TDI.
Liu *et al*., 2017 [43]	Ecological study	USA (2004–2009)	Smoke waves (high-pollution episodes from wildfire smoke)	Hospitalizations for respiratory illness (COPD and respiratory tract infections)	Females had a higher risk of hospital admissions than males on smoke-wave days vs non-smoke-wave days, 10.4% and 3.7%, respectively.	NA
Majeed *et al*., 2021 [54]	Ecological study	USA, Canada and UK (2000–2017)	SST variability	AMI or IDH admissions and deaths	A shift from cool to warm of ENSO was associated with reduced AMI admissions in Canada (adjusted RR 0.89; 95% CI: 0.80–0.99). AMO during strong positive phase was associated with reduced AMI admissions in eastern Canada (RR 0.93; 95% CI: 0.87–0.98).	A shift from cool to warm of ENSO was associated with increase AMI deaths in USA (RR 1.09; 95% CI: 1.04–1.15). AMO during strong positive phase was associated with increased IHD deaths in the UK (RR 1.08; 95% CI: 1.03–1.14).
McTavish *et al*., 2018 [44]	Case-control study	Canada (2005–2012)	Heat periods (3 consecutive days)	Hospital encounters for AKI	Heat periods were associated with a higher risk for AKI (adjusted OR, 1.11; 95% CI: 1.00–1.23).	NA
Pearce *et al*., 2016 [55]	Ecological study	Australia (1999–2006)	T variability (short-term T patterns: 1–30 days)	Nonaccidental all-cause mortality	NA	Mortality risk increased by 6% when daily average T was higher (above 90^th^ percentile (≥21°C) vs 25-75^th^ (11–17°C)). Mortality increased under higher T with increasing trajectories (T rising).
Petkova *et al*., 2021 [45]	Ecological study	Bulgaria (2000–2017)	Non-optimum T (25-day lag)	All-cause mortality	NA	12.3% (95% CI: 7.3–16.8) of deaths due to non-optimum T. T-attributable mortality was from moderate cold (10.9%; 95% CI: 5.8–15.6), moderate heat (1.1%; 0.5–1.6), extreme cold (0.6%; 0.4–0.7) and extreme heat (0.3%; 0.1–0.4).
Qiu, *et al*., 2022 [46]	Case-crossover study	USA (2000–2016)	Increased T	Acute psychiatric hospitalizations (depression, schizophrenia, and bipolar disorder)	Each 5°C increase in short-term exposure to cold season T increased the relative risk of hospitalization by 3.66% (95% CI: 3.06–4.26) for depression, 3.03% (2.04–4.02) for schizophrenia and 3.52% (2.38–4.68) for bipolar disorder.	NA
Sagy *et al*., 2016 [47]	Retrospective cohort study	Israel (2006–2011)	High daily T	Renal function impairment (in patients who taking anti-hypertensive medication)	A daily increment of 5°C was associated with creatinine increase (reflecting the change in renal function) among patients in the non-treatment group (baseline creatinine adjusted 0.824 mg/dL, % change 1.212, % change 95% CI: 0.082–2.354) and in the group treated with anti-hypertensive medication (baseline adjusted 1.032 mg/dL, % change 3.440, % change 95% CI: 1.227–5.700) Association of AKI risk and 5°C increment in patients taking anti-hypertensive medication	NA
Shi *et al*., 2016 [48]	Ecological study	USA (2000–2013)	Seasonal mean T and variability	All-cause mortality	NA	A 1°C increase in summer and winter mean T corresponded to an increase of 2.46% (95% CI: 2.33–2.59) and 1.46% (1.42–1.50) in the death rate, respectively. 1°C increase in T variability yielded 3.71% (95% CI: 3.21–4.22) and 0.59% (0.37–0.81) increases in annual deaths in summer and winter, respectively.
Sun *et al*., 2018 [49]	Prospective cohort study	China (1998–2010)	Intraseasonal T variability	Respiratory disease hospitalizations (total respiratory diseases, pneumonia, and COPD)	The HR was 1.20 (95% CI 1.08–1.32) for total incident respiratory diseases, 1.15 (1.01–1.31) for pneumonia and 1.41 (1.15–1.71) for COPD per 1°C change in wintertime T variability.	NA
Tenías *et al*., 2015 [50]	Case-crossover study	Spain (2000–2008)	Strong winds	Hospital admission for hip fracture	Increased risk of hip fracture incidence associated with days with more frequent and/or high-speed winds by 23.3% (95% CI 7.7–41.1) in the Mediterranean climate	NA
Vanasse *et al*., 2017 [51]	Retrospective cohort study	Canada (2001–2011)	Mean daily T increase (lag-day 3 or 7)	Hospitalizations and deaths due HF	The risk of HF events (death or hospitalization) increased by about 0.7% for each decrease of 1°C in the mean daily T in both lag-day 3 (RR 0.994, 95% CI: 0.992–0.996) and lag-day 7 (RR 0.993, 95% CI: 0.991–0.995).	
Wei *et al*., 2019 [10]	Prospective cohort study	USA (2001–2011)	Mean and variability of summer and winter T	Hospitalizations for dementia	An increase in summer mean T was associated with a 12% (95% CI: 1.09–1.15) increase in the risk of hospitalization. There was a significant increase in the risk of hospitalization per 0.5°C increase in variability of summer T (HR = 1.07; 95% CI: 1.05–1.09).	NA
Xu *et al*., 2022 [52]	Retrospective cohort study	China (1998–2018)	Extreme ENSO conditions	Non-accidental all-cause mortality	NA	Extremely low and high levels of ENSO over lags of 0–12 months were associated with increased mortality risks.
Zheng *et al*., 2016 [53]	Ecological study	China (2009–2011)	DTR	ER admissions for cardiovascular disease (all-cause cardiovascular disease, ischemic heart disease and cerebrovascular disease)	A 1°C increase in DTR corresponded to a 1.46% (95% CI: 0.74–2.19) increase in all cardiovascular diseases, a 1.71% (0.21–3.23) increase in ischemic heart disease and a 1.55% (0.19–2.93) in cerebrovascular disease among females (2-day lag effects). A 1°C increase in DTR corresponded to 1.13% (95% CI: 0.11–2.16) increase in all cardiovascular diseases and a 2.13 (0.51–3.77) in cerebrovascular disease among males (4-day lag effects).	NA

AKI = acute kidney injury; AMI = acute myocardial infarction; AMO = Atlantic Multidecadal Oscillation; CI = confidence interval; COPD = chronic obstructive pulmonary disease; DTR = diurnal temperature range; EH = extreme heat; ENSO = El Niño-southern Oscillation; ER = emergency room; HF = heart failure; HR = hazard ratio; IDH = ischemic heart disease; NA = not applicable; OR = odds ratio; PI = prediction interval; RR = relative risk; SST = sea surface temperature; T = temperature(s); TDI = temperature deviation index; USA = United States of America; vs. = versus. (Only statistically significant results were presented)

As the aim of this systematic review was to identify health indicators, the outcomes of each study were analysed and classified as morbidity or mortality (Table 1). Overall, 10 articles [10, 39, 41, 43, 44, 46, 47, 49, 50, 53] described the impact of climate change phenomena only in morbidity, while 6 [40, 42, 45, 48, 52, 55] addressed only mortality, and 3 [38, 51, 54] addressed both indicators.

The main mortality indicator was **all-cause mortality** (n = 7) [38, 40, 42, 45, 48, 52, 55]. These articles illustrated a significant association between climate change phenomena and increased risk of all-cause mortality. Mortality risk increased by 1.55% (95% PI: 0.17–2.95) during high-severity worsening drought periods in the western United States of America (USA), i.e., at least 150 days period where drought conditions were the same or worse than the previous day [38]. A study in Japan also reported a 0.5% (95% CI: 0.1–1.0) increase in mortality per 1-unit increase in the temperature deviation index (TDI) [42]. Also related to temperature, one study found that each 2.5% increase in death rate corresponded to a 1°C increase in summer mean temperature [48]. ENSO variability, the irregular periodic variation of meteorological conditions over the tropic Pacific Ocean, also impacts non-accidental all-cause mortality risks. Extremely low and high levels of ENSO were both associated with increased mortality risks [52].

Meanwhile, one article focused on **cause-specific mortality**. This study investigated the correlation between heart failure-related deaths and daily temperature in Canada. It was reported that the risk of heart failure death or hospitalization increased by about 0.7% for each decrease of 1°C in the mean daily temperature in the previous 3 (RR 0.994, 95% CI: 0.992–0.996) or 7 days (RR 0.993, 95% CI: 0.991–0.995) [51].

From the articles that studied **morbidity**, outcomes such as hospitalizations, emergency department visits or hospital admissions for cardiovascular causes (hypertension, heart disease, stroke, acute myocardial infarction, ischemic heart disease, cerebrovascular disease and heart failure), respiratory causes (respiratory diseases, pneumonia, chronic obstructive pulmonary disease (COPD) and respiratory tract infections), renal causes (acute renal injury and renal function impairment), heat-related illness, acute psychiatric causes (depression, schizophrenia and bipolar disorder), dementia and hip fracture were described.

Interestingly, a research conducted in China from 2002 to 2018 revealed a noteworthy finding. According to the study, there was a reduction in the rates of heart disease, stroke, and hypertension with every 1°C increase in average annual temperature. Specifically, the rates of heart disease decreased by 6% (OR: 0.94; 95% CI: 0.92–0.95), stroke by 5% (OR: 0.95; 95% CI: 0.94–0.97), and hypertension by 3% (OR: 0.97; 95% CI: 0.96–0.97) [39]. On the other hand, a separate study performed in China from 2009 to 2011 that also investigated changes in ambient temperature and **cardiovascular morbidities**, reported an increased relative risk of emergency room admissions for cardiovascular disease when the diurnal temperature range (DTR) increased. In fact, a 1°C increase in DTR corresponded to a 1.46% (95% CI: 0.74–2.19) and 1.13% (95% CI: 0.11–2.16) increase in all cardiovascular diseases among females (2-day lag effect) and males (4-day lag effect), respectively [53].

Regarding **respiratory morbidities**, an USA study revealed that during full drought periods (at least 150 days), respiratory hospital admissions decreased by -1.99% (95% PI: -3.56,-0.38) compared to non-drought periods [38]. Another relevant finding from this article was that the risk of cardiovascular admission and all-cause mortality increased during drought periods vs non-drought periods in geographic locations experiencing less frequent droughts [38]. Thus, the negative health outcome was worse in areas where the climate change phenomenon was not frequent or common. This may be explained due to population acclimatization. In the western USA, the inhalation of fine particulate matter (PM_2.5_) stemming from wildfire smoke also resulted in alterations in respiratory morbidities. In women and men, the risk of respiratory admissions rose by 10.4% and 3.7% correspondingly when comparing smoke wave days to non-smoke days [43].

Concerning **renal causes**, McTavish *et al*., studied the association between heat periods and acute kidney injury in a Northern climate—Canada. The authors found that heat periods were associated with a higher risk of acute kidney injury by 11% [44]. Elevated daily temperatures were also associated with a detrimental impact on kidney function in the population receiving anti-hypertensive medication [47].

Focusing on **mental health**, the findings from the case-crossover study of Qiu *et al*., showed an increase in relative risk of hospital admission by 3.66% (95% CI: 3.06–4.26) for depression, 3.03% (95% CI: 2.04–4.02) for schizophrenia and 3.52% (95% CI: 2.38–4.68) for bipolar disorder for each 5°C increase in short-term exposure to cold season temperature [46]. Dementia was another neurological disorder explored and an increase in summer mean temperature was reported as associated with a 12% (95% CI: 1.09–1.15) increase in the risk of dementia-associated hospitalization. Likewise, a 0.5°C increase in variability of summer temperature was linked to an increased risk for this condition (HR = 1.07; 95% CI: 1.05–1.09) [10].

**Physical injuries** were also investigated. Two distinct regions with contrasting climates in Spain were explored by Tenías *et al*. and the relationship between weather conditions and incidence of hip fracture was studied. Increased risk of hip fracture incidence was associated with days with more frequent and/or high-speed winds by 23.3% (95% CI 7.7–41.1) in a Mediterranean climate. Meanwhile, in the region characterized by an inland climate, the observed difference in the incidence of hip fractures was comparatively lower and did not reach statistical significance [50].

Interestingly, one main report of the selected articles is that forms of ambient temperature variability have a very strong effect on health outcomes. For example, findings from an examination of the relation between seasonal temperature variability and emergency hospital admissions for respiratory diseases reported an HR of 1.20 (95% CI 1.08–1.32) for total incident respiratory diseases, 1.15 (95% CI 1.01–1.31) for pneumonia and 1.41 (95% CI 1.15–1.71) for COPD per 1°C change in wintertime temperature variability [49]. This is particularly interesting because climate changes imply not only the increase of average ambient temperatures, but the frequency of extreme temperatures but also the variability of temperature within days, seasons, and years. In fact, Shi *et al*. found that a 1°C increase in summer and winter mean temperature corresponded to an increase of 2.46% (95% CI: 2.33–2.59) and 1.46% (95% CI: 1.42–1.50) in the death rate, respectively. On the other hand, a 1°C increase in temperature variability yielded a 3.71% (95% CI: 3.21–4.22) and 0.59% (95% CI: 0.37–0.81) increase in annual deaths in summer and winter, respectively [48].

Also, it is important to highlight that non-optimum temperatures, both high and low, contributed to an increased risk of all-cause mortality [40, 42, 45, 48]. A study in Bulgaria between 2000 and 2017 reported that 12.3% (95% CI: 7.3–16.8) of deaths in that period were due to non-optimum temperatures. The temperature-attributable mortality was from moderate cold (10.9%; 95% CI: 5.8–15.6), followed by moderate heat (1.1%; 95% CI: 0.5–1.6), extreme cold (0.6%; 0.4–0.7) and extreme heat (0.3%; 95% CI: 0.1–0.4) [45].

Furthermore, the majority of the selected articles analysed individual covariates or possible effect modifiers on morbidity or mortality. The most common included were **gender**, **socioeconomic status,** and **education level**. Depending on the climate change phenomenon and health outcome studied, females presented a higher or a lower risk compared to men. For example, in contrast to men, in the findings of Shi *et al*., women were more sensitive to increased summer mean temperature but less to increased season temperature variability [48]. Contrarily, a low socioeconomic status was frequently associated with a higher health risk. A low individual socioeconomic status or poverty was correlated with an increased risk of dementia hospitalization related to a higher mean winter temperature and increased temperature variability [10]. The effect of ENSO in increasing mortality risk was also higher in families who had a low income per capita [52]. In the same way, a low education level was associated with the aggravation of health outcomes. For instance, the relative risk of respiratory admissions on smoke-wave days compared with non-smoke-wave days was higher for people living in areas with less educated individuals (RR = 1.13, 95% CI: 0,97–1.31) comparing to those living in areas with higher educated individuals (RR = 1.06, 95% CI:0.98–1.14) [43].

Importantly, effect modification by different **age subpopulations** among older adults was also frequently considered. The most relevant findings were the following. Younger age groups (65–74 and 75–84 vs >84) were at higher risk for hospitalization due to dementia relating to a higher mean winter temperature or increased temperature variations [10]. Individuals aged 78 years or older had an increased risk of renal/heat/respiratory hospitalizations 6 days following extreme heat compared to individuals aged 65–77 years, 15% (95% CI: 11–19) and 7% (95% CI: 4–10), respectively [41]. Individuals aged 75 or over showed a higher all-cause mortality risk to changes in 1°C in seasonal mean temperature [48]. Finally, individuals aged 85 and over, compared to the 65–84 age group, had a greater burden of temperature-related mortality due to cold or hot temperatures [45].

## Discussion

Although the impact of climate change on human health is widely acknowledged, it remains difficult to attribute morbidity and mortality to the changing climate. Nevertheless, we showed in this systematic review growing evidence that climate change is largely affecting the physical and mental health of older adults (65 years or over).

Based on the existing literature, climate change embraces different manifestations such as climate variability and its frequency and extreme events. Alterations in temperature, heatwaves, precipitation, ENSO and other modes of variability, wildfires, droughts, hurricanes, and floods were already observed and are expected to be aggravated in the future [56, 57]. The present systematic review, covering 19 original studies, revealed that the majority of these climate change phenomena are associated with an increased risk of all-cause and cause-specific mortality and several morbidity outcomes in older adults. The impact on morbidity had several causes, the most common causes of hospitalization were cardiovascular or respiratory disease. Climate changes affect the health of older adults directly through ambient temperature variability, extreme and abnormal temperatures, strong winds, sea temperature variability, extreme ENSO conditions and droughts, and indirectly by air pollution from wildfires. In particular, full drought periods, extreme heat, smoke waves resulting from wildfires, and variations in temperature within a season have an impact on respiratory morbidities. The increase in average annual temperatures, variability in sea surface temperatures, and an overall rise in daily temperatures influence cardiovascular morbidities. Renal morbidities are affected by extreme heat, periods of high temperature, and elevated daily temperatures. Mental health is influenced by temperature increases and variability during summer and winter. Lastly, physical injuries are linked to sudden variations in wind speed or direction.

The higher vulnerability of the older population to heat or cold exposure and other weather events can be attributed to the physiological and pathological changes of age. Aging is associated with impaired thermoregulatory control, which includes a decrease in the capacity to produce sweat, the primary mechanism for heat loss. In fact, older adults experience a progressive variability in body temperature, rendering them less adept at adapting to fluctuations in external temperatures [58]. Aging also compromises the flexibility of the immune system, thus older adults are less responsive to environmental stressors [59]. Renal function impairment is likewise associated with age, as the kidney goes under age-related anatomic and functional changes, such as decreased renal blood flow. Under environmental stress, the aged kidney requires more time to respond to and correct the abnormality [60]. Finally, the presence of multiple comorbidities that may implicate excessive polypharmacy, the reduction of mobility, cognitive limitations and the presence of frailty exacerbate this vulnerability. Social factors, e.g. limited access to care, living alone and isolated and housing without air conditioning systems, can also be implicated [61].

It was possible to observe different effects of climate change phenomena between gender in several health outcomes found. Biological differences and care-seeking behaviours can partly explain these differences. For instance, women may face a greater vulnerability compared to men due to their higher subcutaneous fat and surface-to-mass ratio [62]. Women are also reported to visit care providers for both physical and mental health concerns to a greater extent than men [63]. In addition to examining gender disparities, numerous studies investigate the impact of varying income and education levels on the effects of climate change. Lower socioeconomic status or poverty can exacerbate the consequences of climate change, primarily due to limited access to essential resources such as air conditioning, ventilation systems, and adequate home insulation necessary for maintaining optimal or safe body temperatures. Moreover, individuals in lower socioeconomic groups may also face additional challenges associated with other health conditions and reduced access to healthcare services. Lower levels of education and income can lead to misinformation and less preventive and/or protective behaviours [64]. Therefore, the less educated and the ones with low socioeconomic status among the older adults are more vulnerable to a changing climate.

As climate change phenomena become more intense and frequent, population acclimatization is necessary to survive [65]. However, human adaptation and temperature tolerance have limitations, especially in the more vulnerable groups. Hence, it is crucial to prioritize the implementation of strategies aimed at minimizing exposure to the effects of climate change [65].

Afar the impact on the quality of human life and risk of death, climate change also leads to a high economic burden. Although most available economic evidence on health costs related to the impact of climate change has gaps or limitations, it is clear that implementing health protection measures will yield economic benefits through reduced expenditure on future health treatments [66].

Our review had several strengths. To our knowledge, to date, this is the first systematic review that explores all potential global climate change scenarios and their corresponding health outcomes in individuals aged 65 or older. Also, the literature search was run in three different databases. However, some limitations must be noted. The time frame used can be a limitation in our study. It’s possible that this limited period might not fully capture certain health outcomes more prevalent in older adults. By excluding studies that included mixed populations, to minimize the extent of our analysis, some important health outcomes with more predominance in older adults were not mentioned. Also, only English language studies were considered. Finally, most of the articles included are from developed countries, which may underestimate the burden of climate change. Since developing countries may experience more intense phenomena. Thus, the articles presented in this review may not be a true representation of all valid articles–selection bias.

## Conclusions

This systematic review examined 19 studies that sought to identify health indicators illustrating the influence of climate change on the health and well-being of older adults. The review found various morbidity outcomes, including diseases related to the cardiovascular, respiratory, and renal systems, mental health conditions, and physical injuries. Additionally, the review explored all-cause mortality as well as cause-specific mortality.

Given the unpredictable nature of climate change’s impact on human health, adjusting to its fluctuations poses significant challenges. We hope with this review to provide additional evidence on health indicators in older adults to enable the implementation of more robust prevention and intervention strategies and care-seeking behaviours to promote better adaptation and mitigation of the effects of climate change.

However, studies that relate climate change to older adults and health are still scarce, and the 19 studies we considered eligible mostly crossed hospital and meteorological databases. This may lead to an incomplete representation of the population and health outcomes and may not provide a comprehensive understanding of the broader population, limiting the generalizability of the results to other populations or regions. In some studies, a lack of a control population was also observed. To overcome these limitations, it would be beneficial the production of studies that combine data from multiple sources to provide an accurate assessment of the health impacts of climate change on older adults.

## Supporting information

S1 Checklist PRISMA 2020 checklist. (DOC)
